# The Mouse Median Nerve Experimental Model in Regenerative Research

**DOI:** 10.1155/2014/701682

**Published:** 2014-08-11

**Authors:** Sara Buskbjerg Jager, Giulia Ronchi, Christian Bjerggaard Vaegter, Stefano Geuna

**Affiliations:** ^1^The Lundbeck Foundation Research Center MIND and Danish Research Institute of Translational Neuroscience DENDRITE, Nordic EMBL Partnership, Department of Biomedicine, Aarhus University, Ole Worms Alle 3, 8000 Aarhus C, Denmark; ^2^Department of Clinical and Biological Sciences, Neuroscience Institute of the “Cavalieri Ottolenghi” Foundation (NICO), University of Turin, 10 Regione Gonzole, Orbassano, 10043 Turin, Italy

## Abstract

Sciatic nerve crush injury in rat animal model is one of the most common experimental models used in regenerative research. However, the availability of transgenic mouse for nerve regeneration studies is constantly increasing and, therefore, the shift from rat model to mouse model is, in some cases, necessary. Moreover, since most of the human nerve lesions occur in the upper limb, it is also advantageous to shift from sciatic nerve to median nerve. In this study we described an experimental model which involves lesions of the median nerve in the mouse. Data showed that the finger flexor muscle contraction strength, assessed to evaluate the motor function recovery, and reached values not different from the control already 20 days after injury. The degree of nerve regeneration evaluated with stereological methods in light microscopy showed that, 25 days after injury, the number of regenerated myelinated fibers was comparable to the control, but they were smaller with a thinner myelin thickness. Stereological analysis made in electron microscopy confirmed these results, although the total number of fibers quantified was significantly higher compared to light microscopy analysis, due to the very small size of some fibers that can be detected only in electron microscopy.

## 1. Introduction

The rat sciatic nerve crush model is the most frequently used model in the peripheral nerve regeneration field [1–4]. The preference for the rat compared to the mouse model seems mainly to be due to the rat's greater size which makes the operational procedures less difficult [5]. However, the mouse animal model has become increasingly popular due to the option of transgenic mice, which makes it possible to study the molecular aspects of nerve regeneration in a complex* in vivo* model. In the last few decades, works regarding the use of wild type mouse sciatic nerve [6–8] and transgenic mouse sciatic nerve [9–11] have been published.

Concerning the nerve model used in experimental research, more interest has been recently centered on the use of the median nerve, since many peripheral nerve injuries in humans are seen in the upper limb and therefore the median nerve may be a more clinically relevant model. The median nerve is, together with the ulnar and the radial nerve, one of the major nerves in the forelimb of the mouse. The median nerve predominantly innervates the finger flexor muscle without detectable interference from the ulnar nerve [5] as it is also seen in the rat [12]. This condition makes it possible to evaluate the functional regeneration of the median nerve with the grasping test since it demonstrates the function of the finger flexor muscle [5]. Moreover, the use of the median nerve gives a better animal welfare postsurgically; in recent years, the use of this experimental model has increased, although it is still not widely used in the nerve regeneration research field [5, 13–17].

In this study we describe an experimental model which involves a crush injury of the median nerve in the mouse [15]. The crush lesion was performed with a nonserrated clamp that applies a predetermined force. By keeping the force and the duration of the application constant, it is possible to create a reproducible and homogeneous crush injury [15, 18–21]. The functional recovery was assessed by the grasping test, whereas the degree of regeneration was evaluated with unbiased stereological methods made in both light and electron microscopy.

## 2. Standardized Crush Median Nerve Injury

All mouse experiments were performed with the approval of the local Institution's Animal Care and Ethics Committee of the University of Turin and in accordance with the European Communities Council Directive of 24 November 1986 (86/609/EEC). All efforts were made to minimize both the number of animals used and animal suffering. Experiments were performed on adult female mice. Animals were housed in cages in a temperature-controlled vivarium with* ad libitum* access to food and water. Mice were maintained under standard laboratory conditions with a 12:12 h light/dark cycle.

For the surgery, mice (*n* = 5) were deeply anaesthetized via intraperitoneal injection with ketamine (9 mg/100 g-body weight), xylazine (1.25 mg/100 g-body weight), and atropine (25 *μ*g/100 g-body weight). The skin of the right forelimb was opened in the longitudinal direction proximal to the elbow with a small incision with a scalpel. The muscle layers were separated with blunt dissection and the right median nerve was exposed from its origin at the brachial plexus to the elbow. The nerve was transected at the middle third of the brachium and the proximal stump was sutured to the pectoralis major muscle to avoid reinnervation. This was done to prevent interference from the right forelimb when performing the grasping test. The transected right median nerve was collected and utilized as control nerve. Finally, the skin was closed with surgical suture (5/0 silk suture) and the mouse was allowed to recover.

Two weeks after the first surgery, the animal was anaesthetized, and the left median nerve was exposed and crushed with a nonserrated clamp (manufactured by the Institute of Industrial Electronic and Material Sciences, University of Technology, Vienna, Austria) applied to the middle brachial third of the nerve for 30 seconds with a force of 63.1 N and a final pressure of 20.43 MPa to the nerve [15, 18]. Finally, the skin was closed with surgical suture and the mouse was allowed to recover.

## 3. Functional Assessment of Nerve Regeneration: Grasping Test

Animals were tested before the second operation to estimate the maximum force of the left forelimb and every 5 days after the operation. Grasping test was performed using the BS-GRIP Grip Meter (2 Biological Instruments, Varese, Italy), which consists of a precision dynamometer connected to a grid. The mouse was held by its tail and was placed close enough to reach the grip meter; it was allowed to grasp the grid of the grip meter and lifted away from the grip meter until it lost its grip. The grip meter records the maximum weight the mouse is able to pull. Each mouse was tested three times and the average weight for each mouse was recorded.

**Figure 1:**
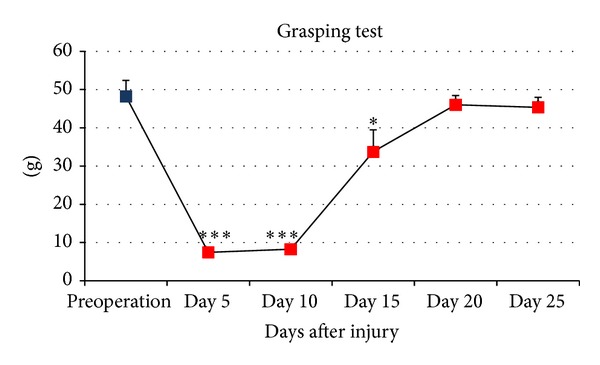
Grasping test. The data from the grasping test show the functional recovery after the mouse median nerve crush injury. Data are reported as means ± standard deviation of five samples in each group. Statistical analysis was performed using paired* t*-test (using SPSS 20.0 software). *P ≤ 0,05; **P ≤ 0,01; and ***P ≤ 0,001 versus preoperation value (control value).

Motor functional analysis results (Figure 1) showed that, from the day of injury to day 10 after injury, the mice were not able to grip the grid because the distal part of the median nerve was still degenerating; the fingers of all the mice were in complete extension, but the values did not fall to zero because the weight of the mouse paw was recorded by the balance. Starting from day 10 the value increased reaching values not significantly different from the preinjury value already at day 20 after injury.

## 4. High-Resolution Light Microscopy Stereological Analysis

25 days after the injury, the median nerve was harvested. Briefly, the mouse was anesthetized via intraperitoneal injection with ketamine (9 mg/100 g-body weight), xylazine (1.25 mg/100 g-body weight), and atropine (0.025 mg 25 *μ*g/100 g-body weight) and the regenerated median nerve (left median nerve) was exposed. A segment of 5 mm distal to the crush site was removed. A 6/0 stitch was used to mark the proximal end of the removed nerve segment. The animal was finally sacrificed by lethal injection of the anesthetic solution. The nerve segment was first fixed for a few seconds in a small drop of fixative solution (glutaraldehyde 2.5% and 0.5% sucrose in 0.1 M Sörensen phosphate buffer, pH 7.4) to maintain the nerve straight in order to sustain specimen's orientation; then, the specimen was placed in an Eppendorf tube with the fixative solution for 6–8 hours.

The sample was washed in 0.1 M Sörensen phosphate buffer with 1.5% sucrose for at least 15 min. The nerve sample was then placed in 2% osmium tetroxide in 0.1 M Sörensen phosphate buffer for 2 hrs and then dehydrated carefully in at least five passages of 5 min in ethanol from 30% to 100%. Two passages of each 7 min in propylene oxide at room temperature were performed followed by one passage of 1 hr in a 1 : 1 mixture of propylene oxide and Glauerts' mixture of resin (0.5% of dibutyl phthalate in a 1 : 1 mix of Araldite M and Araldite M hardener 964) at room temperature. Finally, the sample was embedded in Glauerts' mixture of resin with 2% of the accelerator DMP-30 and was incubated.

The resin-embedded nerves were cut with an Ultracut UCT ultramicrotome (Leica Microsystems, Wetzlar, Germany) with a thickness of 2.5 *μ*m starting from the distal end of the median nerve and stained with 1% Toluidine blue in 1% borax for 30–45 sec while on a hot plate.

One 2.5 *μ*m section from each nerve was selected for light microscopic analysis. Photomicrographs were taken using a DM4000B microscope equipped with a DFC320 digital camera (Leica Microsystems, Wetzlar, Germany) and slightly adjusted for brightness and contrast to obtain uniform plates.

**Figure 2:**
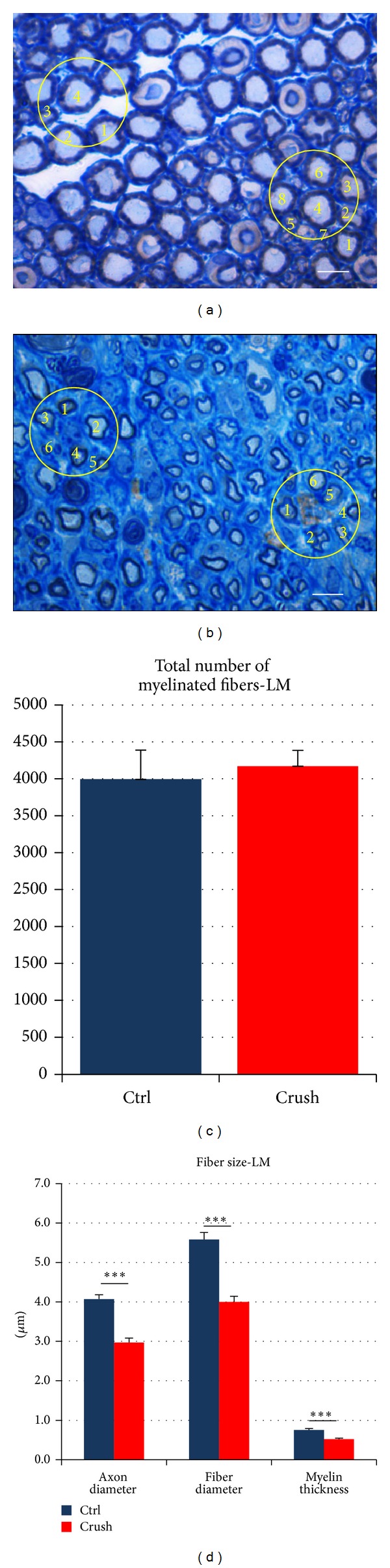
High-resolution light microscopy. (a)-(b) Representative images of toluidine-blue-stained transverse-sectioned control median nerve (a) and regenerated median nerve 25 days after the crush injury (b). The pictures include two circular frames where the myelinated fibers are counted. The yellow numbers indicate the myelinated fibers that have the top inside the circular frame and therefore are counted. Scale  bar = 5 *μ*m. (c) and (d) illustrate the data obtained with the light microscopic stereological analysis. (c) shows the total number of myelinated fibers in the control group and 25 days after the crush injury, while (d) shows the size parameters (axon diameter, fiber diameter, and myelin thickness) in the two experimental groups. Data are reported as means ± standard deviation of five samples in each group. Statistical analysis was performed using paired* t*-test (using SPSS 20.0 software). *P ≤ 0,05; **P ≤ 0,01; and ***P ≤ 0,001.

From the chosen section, the total cross-sectional area of the whole nerve was measured and the quantitative analysis with modified 2D-disector design based sampling was performed. Briefly, 10–15 sample fields from each section were taken as images through a 100x oil-immersion objective (the first image was taken at a random place and the next images were taken based on a fixed distance from the preceding image). Axons were counted in two circular frames in each image (Figures 2(a) and 2(b)). In each sampling field, the “edge effect” was avoided by employing a two-dimensional dissector procedure which is based on sampling the “tops” of the fibers [22, 23] (Figures 2(a) and 2(b)). For each fiber that was counted, the axon and the fiber area were measured. From these measurements the mean fiber density (by dividing the number of myelinated fibers within the circular frames by the areas of the circular frames, fibers/mm^2^) and the total number of nerve fibers (by multiplying the mean fiber density with the total cross-sectional area of the whole nerve) were estimated. Moreover, the circle-fitting diameter of the axon (*d*) and of the fiber (*D*), the myelin thickness ((*D* − *d*)/2), and the *g*-ratio (*D*/*d*) were calculated.

Figures 2(c) and 2(d) show the stereological results obtained with light microscopy analysis. In particular, Figure 2(c) illustrates the total number of myelinated fibers of uninjured nerves (Ctrl) compared with regenerated nerves (crush, 25 days after the injury). No statistical differences are seen between the two groups, in accordance with Ronchi et al., 2010 [15]. In Figure 2(d) the size parameters (axon diameter, fiber diameter, and myelin thickness) of both uninjured nerves and regenerated nerves are shown. As expected, regenerated nerves showed smaller axon and fiber diameter and thinner myelin thickness. Finally, *g*-ratio was not statistically different between the two experimental groups (data not shown).

## 5. Electron Microscopy Stereological Analysis

Electron microscopy analysis was performed on the same specimens used for high-resolution light microscopy. Ultrathin sections (70 nm thick) were cut immediately after the series of semithin sections with the same ultramicrotome and collected on a pioloform coated grid. Sections were finally double-stained with saturated aqueous solution of uranyl acetate and lead citrate. Ultrathin sections were analyzed using a JEM-1010 transmission electron microscope (JEOL, Tokyo, Japan) equipped with a Megaview-III digital camera and a Soft-Imaging-System (SIS, Munster, Germany).

**Figure 3:**
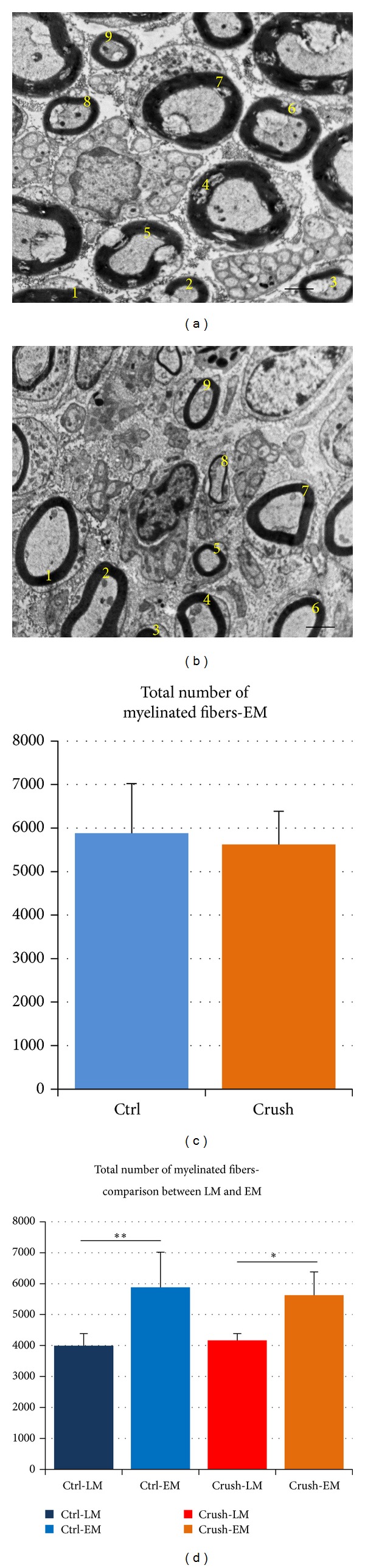
Electron microscopy. (a)-(b) Representative electron micrographs of the control median nerve (a) and regenerated median nerve, 25 days after the injury (b), stained with uranyl acetate and lead citrate. The counting of the myelinated fibers is illustrated with yellow numbers. Scale  bar = 2 *μ*m. (c) Shows the data obtained with the electron microscopic (EM) analysis. It shows the total number of myelinated fibers in control nerves and in regenerated nerves. (d) Comparison of stereological data from light microscopy (LM) and electron microscopy (EM). The figure illustrates that it is possible to count more myelinated axons with the electron microscope compared to the light microscope, in both control (Ctrl) and regenerated group (crush). Data are reported as means ± standard deviation of five samples in each group. Statistical analysis was performed using paired* t*-test (using SPSS 20.0 software). *P ≤ 0,05; **P ≤ 0,01; and ***P ≤ 0,001.

At electron microscopy, an adequate number of fields were selected using a systematic random sampling protocol in live images at 8000x magnification. The number of myelinated fibers was quantified in a defined area (the whole screen that at 8000x is 12.2 × 16.2 *μ*m^2^) applying the same rules as for light microscopy (if the top of the axon is in the live image then the axon is counted and vice versa; Figures 3(a) and 3(b)). Mean fiber density was then calculated by dividing the total number of fibers within the sampling field (N) by its area (N/mm^2^). The total number of fibers was estimated by multiplying the mean fiber density by the total cross-sectional area of the whole nerve cross section which was measured in the light microscopic analysis.

Figure 3(c) shows the stereological results obtained with electron microscopy analysis on the same samples used for light microscopy analysis. No statistical differences were seen in the total number of myelinated fibers between the two experimental groups.

Interestingly, in Figure 3(d) the difference in the total number of myelinated fibers counted in light microscopy or electron microscopy in both controls and regenerated nerves can be appreciated. This difference is due to the different resolutions of the two morphological techniques, since the same nerves are counted with both methods. Indeed, light microscopy underestimates the number of fibers compared to the electron microscopy in both experimental groups.

## 6. Discussion and Conclusions

The use of mice for experimental nerve regeneration investigation is getting more and more popular and both the sciatic and median nerves have been used in several recent research papers [5–8, 13–15, 17]. The methods can be used to investigate the regeneration of the peripheral nervous system on a functional, morphological, and molecular level. Further, it can be applied to transgenic mice to test drugs for any influence on regeneration in regular mice and in other rodents such as rats [9–11, 13, 14, 16].

In this paper we have described a standardized crush protocol that induces a fast regeneration, approximately 20 days on a functional level, which helps keeping expenses low and eases the conduction of the experiment [15]. The fast regeneration can, however, in some cases be a limitation since it can be difficult to achieve a faster functional recovery than seen in the control mice. In this case it might still be possible to see a morphological difference considering the number of regenerating axons obtained by the stereological analysis. An alternative to the crush method is the use of the neurotmesis model (treated by direct end-to-end suture of the two nerve stumps), which has a significantly slower regeneration although it is more challenging from a surgical point of view [4, 5].

A couple of methodological issues deserve special discussion: first, the fixation procedure of nerve specimens that plays a critical role in the histological analysis. In fact, it is important that the nerve has a straight configuration during the fixation in order to be sure that the nerve keeps its orientation making it possible to cut it transversely [24, 25]. Second, although the use of stereological techniques allows obtaining unbiased counts of myelinated nerve fibers, the issue of misidentification of structures should always be kept in mind while interpreting the quantitative results [26]. In particular, the observation that some myelinated axons (usually the smaller ones) are not detectable at light microscope observation leading to a systematic underestimation of the total number of fibers should always be taken into consideration. In selected cases, that is, when the assessment of small myelinated fibers is relevant for the study, the possibility of carrying out counts at electron microscopy should be considered too. Yet, it is interesting to note that stereological methods (especially at electron microscopy level) can also be used to count the number of Schwann cells and other cells in the samples.

In conclusion, we support the view that the mouse median nerve crush model is a valuable tool for the study of posttraumatic nerve damage and regeneration, especially to disclose the cellular and molecular basis of nerve regeneration taking advantage of the large availability of genetically modified mice.
